# Inadequate monitoring risks safety of blood transfusion in rural Zambia

**DOI:** 10.1186/cc14418

**Published:** 2015-03-16

**Authors:** O Todd, K Sikwewa, J Kamp, I Hodt Rasmussen, K Mortensen, S Kudsk-Iversen

**Affiliations:** 1 St Francis Hospital Katete, Zambia; 2 Unversity College Hospital, London, UK

## Introduction

In Zambia, supply of blood is insufficient to meet clinical need, on a national level. Paradoxically, blood is also more often transfused unnecessarily in this setting. The Zambian National Blood Transfusion Service is currently scaling up voluntary blood donation and supply systems, and requires hospitals to improve blood transfusion safety. At a rural district hospital in Zambia, we audited practice and surveyed knowledge amongst staff using standards established in national guidelines.

## Methods

A retrospective audit over 2 months of all blood transfusion forms at St Francis Hospital, Eastern Province, Zambia. Respective patients' notes were reviewed for: record of observations during transfusion; patient demographics; and length of stay. We surveyed nurses' attitudes, confidence and knowledge in relation to blood transfusion standards.

## Results

In May and June 2014, 457 requests were made for blood, of which 157 (34%) received blood transfusion, of which 108 (69%) had records of observations available. The audit demonstrated that requests were mostly complete (90%), but urgency was indicated in only 32%. The matching of blood to patient by more than one nurse was recorded amongst 66% of cases. Only 2% of transfusions met minimal requirements for transfusion reaction monitoring. Of nurses surveyed (*n *= 20), most were experienced in their post (mean 7.3 years, range 2 weeks to 20 years). Nurses rated themselves as highly confident in handling blood transfusions and identifying and dealing with transfusion reactions. However, 90% believed they could identify all transfusion reactions by measuring temperature alone, and 25% would measure temperature only as a parameter to monitor the transfusion, even in ideal settings. Most knew to check observations before, 15 minutes after the start of transfusion and then hourly thereafter (88%); but only 10% would check at the start, at completion and 4 hours after completing transfusion. The most frequently reported reason for not doing observations was time pressure on the ward (85%).

## Conclusion

In this setting, current practice is evidently inadequate to identify and prevent blood transfusion reactions. The survey revealed high confidence but patchy knowledge amongst nurses of the requirements for safe blood transfusion. Better timing to transfuse at times when nursing staff numbers are higher, alongside compulsory training, may together represent potential low-cost interventions to improve blood transfusion safety.

